# Rapid Identification of *Mycobacterium tuberculosis* Complex Using Mass Spectrometry: A Proof of Concept

**DOI:** 10.3389/fmicb.2022.753969

**Published:** 2022-03-31

**Authors:** Simon Robinne, Jamal Saad, Madjid Morsli, Zelika Harouna Hamidou, Fatah Tazerart, Michel Drancourt, Sophie Alexandra Baron

**Affiliations:** ^1^Aix-Marseille-University, IRD, MEPHI, Marseille, France; ^2^Assistance Publique-Hôpitaux de Marseille, Marseille, France; ^3^IHU Méditerranée Infection, Marseille, France; ^4^Laboratoire National de Référence des IST/VIH et de la Tuberculose, Niamey, Niger; ^5^Institut des Sciences Vétérinaires, Université de Blida 1, Blida, Algeria

**Keywords:** *Mycobacterium*, *Mycobacterium tuberculosis*, tuberculosis, MALDI-TOF mass spectrometry, whole genome sequencing, identification

## Abstract

Mycobacteria that form the *Mycobacterium tuberculosis* complex are responsible for deadly tuberculosis in animals and patients. Identification of these pathogens at the species level is of primary importance for treatment and source tracing and currently relies on DNA analysis, including whole genome sequencing (WGS), which requires a whole day. In this study, we report the unprecedented discrimination of *M. tuberculosis* complex species using matrix-assisted laser desorption ionization-time of flight mass spectrometry (MALDI-TOF-MS), with WGS as the comparative reference standard. In the first step, optimized peptide extraction applied to 36 isolates otherwise identified in five of the 11 *M. tuberculosis* complex variants by WGS yielded 139 MALDI-TOF spectra, which were used to identify biomarkers of interest that facilitate differentiation between variants. In a second step, 70/80 (88%) other isolates were correctly classified by an algorithm based on specific peaks. This study is the first to report a MALDI-TOF-MS method for discriminating *M. tuberculosis* complex mycobacteria that is easily implemented in clinical microbiology laboratories.

## Introduction

Mycobacteria that form the *Mycobacterium tuberculosis* complex (MBTC) are responsible for tuberculosis, a deadly infection that is a major public health concern worldwide (Forbes et al., 2018). MBTC is composed of different species that have been recently reclassified as variants of *M. tuberculosis*: *M. tuberculosis* var. tuberculosis, *M. tuberculosis* var. africanum, *M. tuberculosis* var. bovis, *M. tuberculosis* var. BCG, *M. tuberculosis* var. caprae, *M. tuberculosis* var. microti, *M. tuberculosis* var. pinnipedii, *M. tuberculosis* var. canettii, *M. tuberculosis* var. suricattae, *M. tuberculosis* var. orygis, and *M. tuberculosis* var. mungi (Malone and Gordon, 2017; Riojas et al., 2018). In our clinical microbiology practice, however, *M. tuberculosis sensu stricto*, along with *Mycobacterium bovis* and its derivative Bacillus Calmette-Guérin (BCG) and *Mycobacterium africanum*, are the sole MBTC variants that we have encountered for 10 years. For a convenient terminology, we will use the term “species” to distinguish the different variants of the MTBC.

The distinction between *M. tuberculosis* variants is a concern for the immediate medical management of patients, as, for example, *M. bovis* and BCG are two species acknowledged to be pyrazinamide-resistant (Wanzala et al., 2019). This distinction is also of concern in terms of tracing the source to prevent additional cases, such as zoonotic *M. bovis* tuberculosis (Saad et al., 2020).

While real-time PCR detection of MBTC performed directly in clinical samples is now widely used for the routine screening of tuberculosis, this technique does not enable species identification within the complex (Iketleng et al., 2018). In routine clinical microbiology, this identification is achieved by DNA sequencing investigations using commercially available line probe assays (Richter et al., 2003; Pinsky and Banaei, 2008; Shea et al., 2017; Loiseau et al., 2019) and, at best, through whole genome sequencing (WGS) of the colony (Iketleng et al., 2018). These techniques, however, require specific equipment that may not be available at the point-of-care, with results taking a few days to become available.

Matrix-assisted laser desorption ionization-time of flight mass spectrometry (MALDI-TOF-MS) is a widely implemented technique for the routine identification of bacteria in clinical microbiology laboratories. It is a rapid and low-cost method for identifying bacterial species (Seng et al., 2013; Lagier et al., 2017) and is also routinely used for the identification of mycobacteria (Patel, 2015; Alcaide et al., 2018). However, MALDI-TOF-MS suffers from the same limitation as real-time PCR, i.e., the impossibility of differentiating MBTC species based on similar peptide profile matching. Thus, the specific identification of tuberculosis species using this method is currently impossible (O’Connor et al., 2020).

In this study, we investigated the peptidic profile of MBTC isolates to identify relevant biomarkers specific to one or more species that may provide a fast and cost-efficient method of screening. This method is particularly useful for differentiating groups of isolates with similar spectra and is indistinguishable from a non-selective pattern matching method such as biotyping. It relies on the identification of relevant specific peaks in a MALDI-TOF spectral dataset from isolates already identified using another method at the species, subspecies, or variant level to focus on the pattern matching of spectra on these peaks. Previous studies used this approach to distinguish methicillin-resistant *Staphylococcus aureus* clones from non-resistant clones (Camoez et al., 2016; Wang et al., 2018) or different *Streptococcus pneumoniae* serotypes (Nakano et al., 2015) directly in MALDI-TOF spectra. Application of this method to distinguish subspecies of mycobacteria has shown its efficiency on the *Mycobacterium abscessus* complex (Fangous et al., 2014) and on the MBTC in the specific situation of *M. bovis* from veterinary samples (Bacanelli et al., 2019). Hence, the present study is the first to investigate the differentiation of clinically relevant species in MBTC using MALDI-TOF MS and associated software.

## Materials and Methods

### Mycobacterial Isolates

One hundred twenty-eight clinical isolates preserved in the Collection de Souches de l’Unité des Rickettsies (CSUR) and cultured in the BSL3 laboratory of the IHU Méditerranée Infection, Marseille, France were included in this study. This collection of isolates has previously been investigated (Bouzid et al., 2018; Tazerart et al., 2021a,b) in part for accurate identification by WGS, and was used here as the reference standard (Saad et al., submitted for publication). In addition, four isolates of reference strains ordered from other collections were added to this study: *M. bovis* BCG Pasteur 1173P2, *M. bovis* BCG Tokyo 172, *Mycobacterium canettii* CIPT 140010059, and *Mycobacterium gilvum* DSM 45363 (Table 1).

**TABLE 1:** Mycobacterial isolates and associated spectra.

	Number of isolates	Number of spectra	Origin
**Reference group**			
*M. africanum*	3	12	Collection de Souches de l’Unité des Rickettsies (CSUR), Marseille
*M. bovis*	7	25	
*M. bovis* BCG	1	4	Reference strain: BCG Pasteur 1173P2
	3	12	Collection de Souches de l’Unité des Rickettsies (CSUR), Marseille
*M. canettii*	1	3	Reference strain: CIPT 140010059
	2	8	Collection de Souches de l’Unité des Rickettsies (CSUR), Marseille
*M. tuberculosis*	19	75	
Lineage 1 (Indo-Oceanic)	3	12	
Lineage 2 (East Asia)	5	20	
Lineage 3 (East African-Indian)	4	16	
Lineage 4 (Euro-American)	7	27	
**Total**	36	139	
**Negative control**			
*M. abscessus*	2	8	Collection de Souches de l’Unité des Rickettsies (CSUR), Marseille
*M. avium*	2	8	
*M. chelonae*	1	4	
*M. gilvum*	1	4	Reference strain: DSM 45363
*M. persicum*	4	16	Collection de Souches de l’Unité des Rickettsies (CSUR), Marseille
*M. xenopi*	2	8	
**Total**	12	48	
**Test group**			
*M. africanum*	1	4	Collection de Souches de l’Unité des Rickettsies (CSUR), Marseille
*M. bovis*	6	24	
*M. bovis* BCG	1	4	Reference strain: BCG Tokyo 172
	1	4	Collection de Souches de l’Unité des Rickettsies (CSUR), Marseille
*M. canettii*	3	11	
*M. tuberculosis*	68	265	
Lineage 1 (Indo-Oceanic)	4	15	
Lineage 2 (East Asia)	8	31	
Lineage 3 (East African-Indian)	7	27	
Lineage 4 (Euro-American)	49	192	
**Total**	80	312	

In the first phase, 36 isolates identified by WGS, such as *M. tuberculosis*, *M. africanum*, *M. bovis*, *M. bovis* BCG, and *M. canettii*, were defined as reference strains for the identification of potential relevant biomarkers. Sublineages of *M. tuberculosis* isolates were also investigated, as they present relevant information for diagnosis, notably on antibiotic resistance. These isolates were selected as representative tuberculous mycobacterial species encountered in our clinical experience. Indeed, from 2017 to 2020, WGS lineage identification of 218 MBTC strains yielded 8 (3.7%) as *M. bovis*, 4 (1.8%) as *M. africanum*, and 4 (1.8%) as *M. bovis* BCG. Similarly, in the WHO report on tuberculosis in 2020, *M. bovis* infections were estimated to represent 1.47% of MTBC clinical cases worldwide. *M. canettii* strains were added to the work, although no clinical isolate has been encountered in our unit to challenge the method in identifying unusual MTBC species. In addition, 12 non-tuberculous mycobacteria identified using WGS (*M. abscessus*, *Mycobacterium avium*, *Mycobacterium chelonae*, *M. gilvum, Mycobacterium persicum*, and *Mycobacterium xenopi)* served as negative controls for the MALDI-TOF Biotyper identification method (Bruker Daltonics, Bremen, Germany). These isolates were selected as representative non-tuberculous mycobacterial species encountered in our clinical experience.

In the second phase, 80 isolates were defined as our test group to evaluate the reliability of discriminating biomarkers identified in the first phase.

All isolates were cultured on TransBK m4 agar medium (Culture-Top, Marseille, France), a culture medium derived from Middlebrook 7H10 agar medium, at 37°C in a 5% CO_2_ atmosphere for a minimum of 7 days. In addition, 17 isolates selected from the former collection were cultured on Middlebrook 7H10 agar medium (Becton Dickinson Medical, Le Pont-de-Claix, France) and on Coletsos medium (BioRad, Marnes-la-Coquette, France) at 37°C in a 5% CO_2_ atmosphere for a minimum of 7 days to validate the applicability of the method regardless of the growth medium used (Table 2).

**TABLE 2:** Mycobacterial isolates and associated spectra from cultures grown on different media.

	Number of isolates	Number of spectra obtained for isolates cultured on M7H10 medium	Number of spectra obtained isolates cultured on Coletsos medium	Origin
*M. bovis*	2	8	7	Collection de Souches de l’Unité des Rickettsies (CSUR), Marseille
*M. bovis* BCG	1	4	4	
*M. canettii*	1	4	3	Reference strain: CIPT 140010059
	1	4	1	Collection de Souches de l’Unité des Rickettsies (CSUR), Marseille
*M. tuberculosis*	12	47	37	Collection de Souches de l’Unité des Rickettsies (CSUR), Marseille
Lineage 1 (Indo-Oceanic)	2	7	6	
Lineage 2 (East Asia)	3*	12	8	
Lineage 3 (East African-Indian)	3	12	11	
Lineage 4 (Euro-American)	4*	16	12	
**Total**	17	67	52	

*One isolate of M. tuberculosis Lineage 2 and one isolate of M. tuberculosis Lineage 4 were not cultivated on Coletsos medium.

Total DNA extraction and WGS were performed as previously described (Saad et al., submitted for publication; Saad et al., 2019).

### Matrix-Assisted Laser Desorption Ionization-Time of Flight Mass Spectrometry Analysis

Between one and three 1-μl plastic loops harvested from each isolate were mixed with 300 μl of high purity liquid chromatography (HPLC)-grade water in a 1.5-ml tube (Sarstedt, Nümbrecht, Germany). Samples were then heated to 100°C for 30 min to inactivate mycobacteria (Zwadyk et al., 1994). After cooling, 900 μl of ethanol were added, vortexed for 1 min and centrifuged for 2 min at 16,060 × *g*, and the supernatant was removed. The centrifugation step was repeated twice, and the pellet was dried at room temperature for 1 min. A spatula full of glass beads for cell disruption (diameter: 0.5 mm; Scientific Industries Inc., Bohemia, United States) and 20 μl of acetonitrile were added, and then the samples were stirred using a vortex device for 1 min. Twenty microliters of a solution composed of 70% formic acid were then added and agitated for 5 s. Samples were subsequently agitated using a FastPrep-24™ device (MP Biomedicals, Illkirch-Graffenstaden, Germany, #6004500) for five cycles of 20 s at 4.0 m/s, with a resting time of 5 s between each cycle and then centrifuged for 2 min at 16,060 × *g*. Four replicates of 1.5 μl of supernatant were deposited on a MALDI target plate (MSP 96 target polished steel, Bruker Daltonics) and dried at room temperature for 1 min. A matrix solution consisting of 1.5 μl of saturated α-cyano 4-hydroxycinnamic acid (CHCA) (Sigma–Aldrich, Taufkirchen, Germany) in trifluoroacetic acid 2.5% /acetonitrile (1:1) was deposited on each spot and dried for 5 min at room temperature. Two spots consisting of 1.5 μl of CHCA matrix alone were incorporated as negative controls, and two spots consisting of 1.5 μl of a 1/10 diluted solution of Bruker bacterial standard test, an *Escherichia coli* extract spiked with two high-molecular-weight proteins (Bruker Daltonics) mixed with 1.5 μl of CHCA matrix, were deposited on every plate as positive controls. All the solvents used were of MS grade. Mass spectra were obtained using a MicroFlex™ (Bruker Daltonics) MALDI-TOF MS. Spectra were recorded in the mass range from 2,000 to 20,000 Da.

### Spectra Processing and Data Analysis

Spectra were identified using Biotyper Compass Explorer v4.1 (Bruker Daltonics). Each spectrum was compared with the Mycobacteria bead method Library v.3.0 (Bruker Daltonics), MALDI Biotyper Compass IVD Library v9.0, and our in-house database consisting of 52 additional references of mycobacteria from MTBC generated by controlling spectral quality, MALDI-TOF identification, and WGS identification of spectra and isolates used for references as recommended by the manufacturer and several publications (Keys et al., 2004; Saleeb et al., 2011; Rodriguez-Temporal et al., 2017). Spectra were then processed with ClinProTools v2.2 build 78 (Bruker Daltonics): spectra preparation included a baseline subtraction by Top-hat with a 10% minimal baseline width, an exclusion of noisy spectra for which the signal-to-noise ratio (S/N) was inferior at 2, and a smoothing using the Savitsky-Golay method with a width of 2.0 *m/z* for five cycles; finally, all spectra were recalibrated between each other, with an exclusion threshold of 1,000 ppm. Peak picking was then performed on each spectrum with an S/N threshold > 5 and a maximal number of 500 peaks detected. Peaks with similar *m/z* values were aggregated if they presented a minimal occurrence in a single spectra of 25% with a 500 ppm aggregation width. The intensities of peaks were finally calculated by measuring the areas under peaks with a zero-level integration.

In the first phase, spectra were grouped into different classes according to the identification of their corresponding isolate by WGS. Statistical tests such as *t-*test/ANOVA (TTA) and Wilcoxon/Kruskal–Wallis test (WKW) were then used and applied to processed spectra to identify potential class-specific peaks, and the Anderson–Darling test (AD) was used to verify the normal distribution of peak intensities in each class. The quick-classifier (QC) algorithm was calculated based on most discriminating peaks presenting the lowest *p*-value calculated with TTA (PTTA) if possible, i.e., if the intensities of discriminating peaks present a *p* value from the AD test (PAD) > 0.05, confirming their normal distribution, to classify spectra from test group isolates. Otherwise, QC was calculated from peaks presenting the lowest *p* values from the WKW test (PWKW).

## Results

### Speciation of Mycobacteria Based on Database Matching

Matrix-assisted laser desorption ionization-time of flight mass spectrometry spectra presenting a low intensity resulting in a noisy signal were excluded from the study, providing a total of 499 MALDI-TOF-MS spectra for the *Mycobacterium* species investigated (Table 1), with a mean of 3.90 spectra per isolate. Identification of MTBC isolate spectra by comparison with the selected databases yielded 95.3% correct identifications of *Mycobacterium* species with a comparison score greater than 2.00, and this value increased to 99.7% using a comparison score greater than 1.80. All spectra from isolates of the reference and test groups presented a first matching hit with the MBTC database. Identifications of negative control isolates are presented in Table 3. While *M. abscessus*, *M. avium*, *M. gilvum*, and *M. xenopi* presented reliable identification against the Bruker Mycobacteria bead method library, *M. chelonae* and *M. persicum* presented incorrect identifications. *M. chelonae* was incorrectly identified as *Mycobacterium salmoniphilum*, an *M. chelonae*-related species (Whipps et al., 2007), and *M. persicum* presented over 10 spectra with no reliable identification, while six spectra presented the best matching hit for *Mycobacterium kansasii* (Score: 1.97). This result might be explained by the absence of an *M. persicum* reference in the mycobacteria bead method library and the genetic similarity of *M. persicum* and *M. kansasii* (Jagielski et al., 2019).

**TABLE 3:** Negative control isolate identifications compared with the Bruker Mycobacteria beads method MALDI-TOF library.

WGS identification	Best matching hit from Bruker database	Highest score of identification	Number of spectra with a score > 2.0	Number of spectra with a score > 1.8
*M. abscessus*	*M. abscessus*	2,02	1	7
*M. avium*	*M. avium*	2,32	4	7
*M. chelonae*	*M. salmoniphilum*	1,89	0	3
*M. gilvum*	*M. gilvum*	1,9	0	4
*M. persicum*	*M. kansasii*	1,97	0	6
*M. xenopi*	*M. xenopi*	2,12	6	8

### Identification of Specific Biomarkers and Classification Method Calculation

Spectra from the reference group were first separated into eight classes corresponding to their WGS identification (*M. africanum, M. bovis, M. bovis* BCG, *M. canettii, M. tuberculosis* Lineage 1, *M. tuberculosis* Lineage 2, *M. tuberculosis* Lineage 3, and *M. tuberculosis* Lineage 4). Statistical tests on average spectra from each class identified 84 peaks presenting a PTTA and PWKW value < 0.05. Among these 84 peaks, 11 presented a PAD > 0.05. The QC (QC1) was then calculated for the 23 peaks presenting the lowest PWKW values (Supplementary Table 1). QC1 presented a cross-validation rate of only 48% and a predictive recognition capability of 62%, suggesting that it was not sufficiently reliable to correctly classify spectra among different classes. Evaluation of the classification reliability of QC1 was achieved on spectra from isolates of the test group.

QC1 presented a very low rate of correct classification of 17% for these spectra, confirming that the discriminating power of the classifier was insufficient. Somehow, by considering that the classification of one lineage of *M. tuberculosis* as another was correct, QC1 presented a correct classification rate of 74% (Table 4). Thus, QC1 was unable to discriminate correctly between different lineages of *M. tuberculosis.* Moreover, while correct classification rate of spectra from isolates of *M. tuberculosis* Lineages 2, 3, and 4 improved substantially by implementing this criterion (from 23 to 81, from 15 to 85, and from 16 to 88%, respectively), the correct classification rate of *M. tuberculosis* Lineage 1 only improved from 20 to 47%, which was still not sufficient to be considered a correct identification rate. Finally, correct classification rates for classes other than *M. tuberculosis* isolates were very low (*M. africanum*: 0%; *M. bovis*: 25%; *M. bovis* BCG: 13%; *M. canettii*: 9%). Peaks selected by QC1 were therefore investigated to explain these rates.

**TABLE 4:** Correct classification rate of spectra from test group isolates according to QC1 by considering (a) strictly correct classification or (b) different lineages of *M. tuberculosis* as one class.

Corresponding identification of spectra isolate	Number of spectra	Correct classification rate	
		(a).	(b).
*M. africanum*	4	0%	
*M. bovis*	24	25%	
*M. bovis* BCG	8	13%	
*M. canettii*	11	9%	
*M. tuberculosis*	265	17%	85%
Lineage 1 (Indo-Oceanic)	15	20%	47%
Lineage 2 (East Asia)	31	23%	81%
Lineage 3 (East African-Indian)	27	15%	85%
Lineage 4 (Euro-American)	192	16%	88%
**Total**	312	17%	74%

**FIGURE 1:**
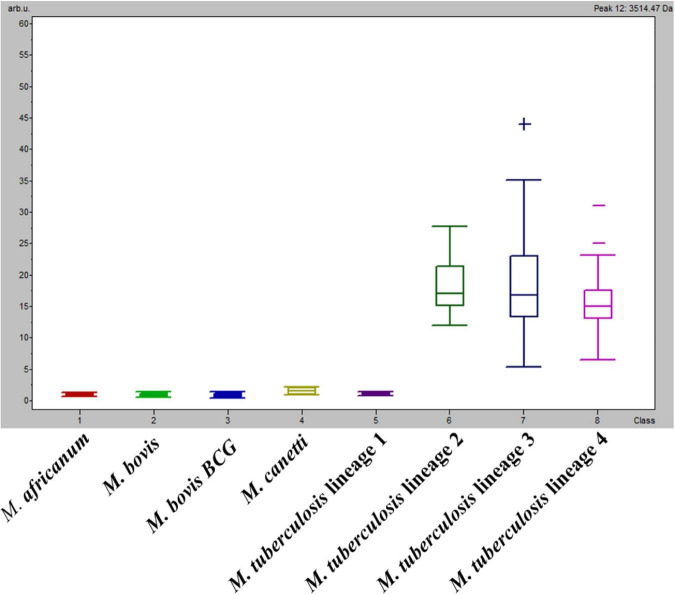
Average intensities and variance (box plot) of *m/z:* 3,514 Da among classes.

One peak measured at *m/z*: 3,514 Da presented low PTTA and PW/KW values (both < 1E^–6^), and the second maximum difference of peak intensity average between classes (DAve) was of particular interest, as it presented an absence/presence discriminatory pattern among classes instead of a difference in the peak intensity ratio (Figure 1). More precisely, the *m/z*: 3,514 Da peak was present in *M. tuberculosis* Lineages 2, 3, and 4 (average peak intensities of 20.1, 21.2, and 17.3 a.u., respectively) and absent from *M. africanum*, *M. bovis* and *M. bovis* BCG, *M. canettii*, and *M. tuberculosis* Lineage 1 (all average peak intensities < 1.5). This finding might explain the low rate of classification of *M. tuberculosis* Lineage 1 spectra. Hence, an approach focusing on this biomarker was investigated.

### Discriminatory Power of the Identified Biomarker at *m/z*: 3,514 Da

**FIGURE 2:**
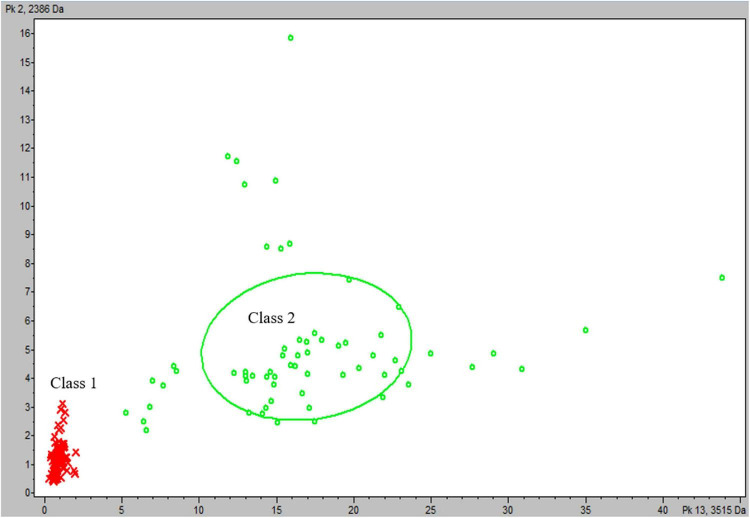
Scatter plot of reference group spectra from classes according to the most discriminating peaks at *m/z*: 3,515 and 2,386 Da. Class 1 (*M. africanum*, *M. bovis*, *M. bovis* BCG, *M. canettii*, and *M. tuberculosis* Lineage 1): red cross and Class 2 (*M. tuberculosis* Lineages 2, 3, and 4): green circle.

Spectra from the reference group were divided into two classes according to observations of *m/z*: 3,514 Da peak intensities: the first class contained spectra from *M. africanum*, *M. bovis* and *M. bovis* BCG, *M. canettii*, and *M. tuberculosis* Lineage 1 isolates (*n* = 76), and the second class contained spectra from *M. tuberculosis* Lineages 2, 3, and 4 (*n* = 63). Statistical tests on average spectra from each class identified 37 peaks presenting a PWKW value less than 0.05. Five of these peaks, including the 3,515 Da peak, were selected by the QC algorithm calculated (QC2) as being more relevant for the classification of spectra (Supplementary Table 2 and Figure 2). QC2 presented a cross-validation rate of 99% and a recognition capability of 100%. The QC2 evaluation of the test group yielded an overall correct classification rate of 89%, with correct spectra classification rates of 100% for Class 1 and 86% for Class 2. The relevance of QC2 in terms of the classification of isolates was evaluated, and a classification was considered as determined for each isolate if half or more spectra acquired from this isolate shared the same classification. Based on these criteria, QC2 presented an overall correct isolate classification rate of 87.5% (70/80), divided into 100% correct classification for Class 1 (16/16) and 84% (54/64) for Class 2, with four isolates belonging to this class according to the incorrectly classified WGS identification (5%) and six isolates with an undetermined classification (7.5%) (Table 5). Thus, metrics such as accuracy (Acc), sensitivity (Se), specificity (Sp), and predictive positive value (Vpp) of QC2 were calculated by considering mycobacteria isolates belonging to Class 2 (*M. tuberculosis* Lineages 2, 3, or 4) that were correctly classified as true positives (TPs) and mycobacteria isolates belonging to Class 1 (*M. africanum/M. bovis/M. bovis* BCG/*M. canetti/M. tuberculosis* Lineage 1) that were correctly classified as true negatives (TNs), as mycobacteria isolates that were incorrectly classified as Class 1 as false positives (FPs) and mycobacteria isolates that were incorrectly classified as Class 2 as false negatives (FNs). Acc and Se were reported as ranges by counting undetermined classifications in either TPs or FNs to consider undetermined classification results for Class 1 isolates. Therefore, metrics of QC2 on the confirmation of the belonging of an MBTC isolate as being part of *M. tuberculosis* Lineages 2, 3, or 4 were as follows: Acc: 87.5–95%; Se: 84–94%; Sp: 100%; and Vpp: 100%.

**TABLE 5:** Correct classification rates for spectra and isolates from test group isolates according to QC2.

Classes	Correct spectra classification rate		Correct isolate classification rate	
**Class 1**: *M. africanum/M. bovis/M. bovis* BCG/*M. canetti/M. tuberculosis* Lineage 1 (Indo-Oceanic)	62	100%	16	100%
**Class 2:** *M. tuberculosis* Lineage 2 (East Asia)/3 (East African-Indian)/4 (Euro-American)	216	86%	54	84%
**Total**	278	89%	70	87,5%

### Evaluation of the Effect of Growth Media

Among 17 isolates cultured in parallel on Middlebrook 7H10 (M7H10) and Coletsos media, 17 and 15 presented satisfying growth after 7 days on M7H10 and Coletsos media, respectively. Indeed, one isolate identified by WGS as *M. tuberculosis* Lineage 2 (East Asia) and one isolate identified by WGS as *M. tuberculosis* Lineage 4 (Euro-American) presented insufficient culture on Coletsos medium. One hundred twenty-eight spectra were generated for these isolates using the same experimental procedure as mentioned above. Then, nine spectra were excluded by ClinProTools, as they presented a low signal (S/N < 2). The remaining 119 spectra were classified using the QC2 algorithm. QC2 presented a correct classification accuracy between Class 1 (*M. africanum*/*M. bovis*/*M. bovis* BCG/*M. tuberculosis* Lineage 1) and Class 2 (*M. tuberculosis* Lineages 2/3/4) of 94.0% (63/67) for spectra from isolates grown on M7H10 medium and 88.5% (46/52) for spectra from isolates grown on Coletsos medium. In terms of isolates, QC2 presented a correct isolate classification rate of 94.1% (16/17) and one isolate with an undetermined classification after culture on M7H10 growth medium, and 86.7% (13/15) and two isolates with an undetermined classification after culture on Coletsos growth medium (Table 6).

**TABLE 6:** Correct classification rates for spectra and isolates from isolates grown on other culture media according to QC2.

Growth medium used	M7H10				Coletsos			
Classes	Correct spectra classification rate		Correct isolate classification rate		Correct spectra classification rate		Correct isolate classification rate	
**Class 1:** *M. africanum/M. bovis/M. bovis* BCG*/M. canettii/M. tuberculosis* Lineage 1 (Indo-Oceanic)	27	100%	7	100%	21	100%	7	100%
**Class 2:** *M. tuberculosis* Lineage 2 (East Asia)/3 (East African-Indian)/4 (Euro-American)	36	90%	9	90%	25	81%	6	75%
**Total**	63	94%	16	94%	46	88%	13	87%

**FIGURE 3:**
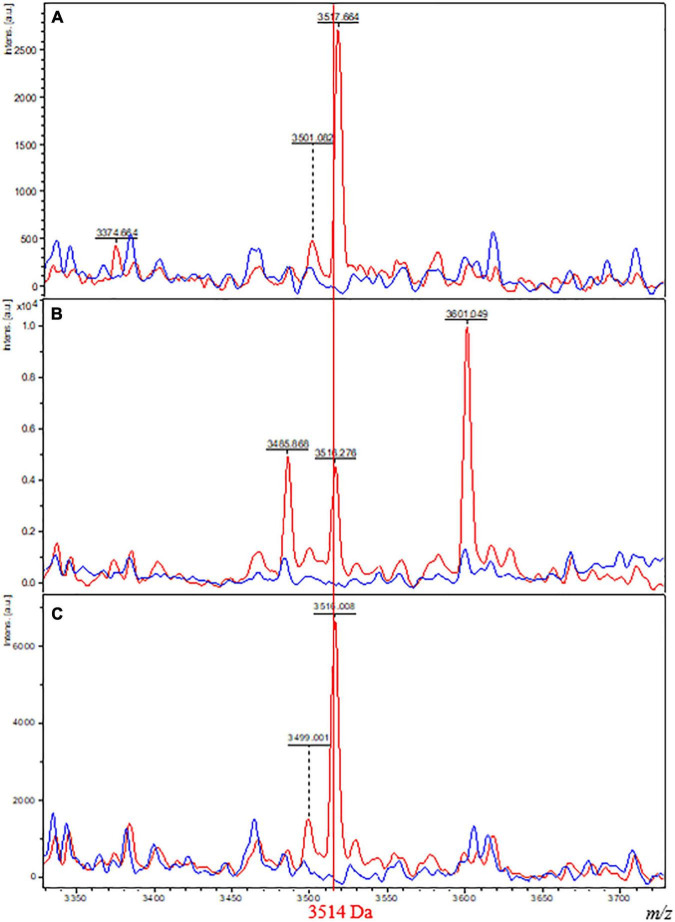
Comparison of MALDI-TOF spectra plots from isolates of Class 1 and 2 grown on different culture media. Spectra of one isolate belonging to Class 1 (*M. tuberculosis* Lineage 2, red) and one isolate belonging to Class 2 (*M. bovis* BCG, blue) grown on M4 agar **(A)**, M7H10 **(B)**, and Coletsos **(C)** media. The *x*-axis shows the *m/z* values on a reduced window of 350 Da comprising the specific biomarker of interest at 3,514 Da. The *y*-axis indicates the intensity of peaks in arbitrary units.

As expected from these results, direct observation of the 3,514-Da biomarker using FlexAnalysis software (Bruker Daltonics) resulted in the observation of the presence of a peak at 3,514 Da for spectra from isolates belonging to Class 2 and an absence of this peak for spectra from isolates belonging to Class 1 (Figure 3).

## Discussion

In this study, we report the unprecedented discrimination of MBTC species using the MALDI-TOF-MS approach. We showed that MALDI-TOF-MS spectra of mycobacteria can be used as a first-line method for screening isolates in the MBTC, presenting a reasonable sensitivity of 93% (84–94%) and a specificity of 100% for confirming the identification of *M. tuberculosis* belonging to Lineages 2 (East Asia), 3 (East African-Indian), or 4 (Euro-American). However, the specificity metric was less reliable than the sensitivity, as only 16 of the 80 isolates from the test group were considered negative for this test. Confidence in this metric might be increased by increasing the number of additional members (*M. africanum, M. bovis, M. bovis* BCG, and *M. tuberculosis* Lineage 1) of the MTBC in the test group. However, these species are still not easily affordable in large quantities. The American Type Culture Collection (ATCC), for example, currently proposes in its collection a total of 69 *M. tuberculosis* isolates, including only 34 *M. bovis*, 2 *M. africanum*, and no *M. canettii*.

These results have been consolidated using several controls that include positive (*E. coli* with a score greater than 2.00) and negative controls (no identification with a score less than 1.60) deposited on each MALDI-TOF-MS target plate; the acquisition of MALDI-TOF-MS spectra at least four times; and the identification of isolates using WGS as the gold standard. Finally, spectra from the reference group were deposited in a publicly available database maintained at our Institute^1^,^2^.

The method presented here would be improved by increasing the number of spectra for the reference group to obtain a more accurate classifier algorithm, especially for members of the MBTC rarely encountered in clinical human diagnosis, such as *M. africanum* and *M. canettii*, which represented four and six isolates of the entire cohort of 116 isolates between the reference and test groups, respectively. Additional data may achieve classification between variants of the Class 1 group (*M. africanum*, *M. bovis*, *M. bovis* BCG, *M. canettii*, and *M. tuberculosis* Lineage 1). Distinction between Lineages 2, 3, and 4 of *M. tuberculosis* has not been achieved using this method, confirming results reported in a previous study by O’Connor et al. (2020) suggesting that this discrimination was currently impossible with a MALDI-TOF-MS data-based approach. Somehow, the data presented here highlighted a relevant difference between *M. tuberculosis* Lineage 1 (Indo-Oceanic) and other lineages, as spectra from isolates of this lineage did not present the biomarker investigated. Interestingly, Brosch et al. documented that *M. tuberculosis* Lineages 2, 3, and 4, which are referred as “modern” strains of *M. tuberculosis*, differed from *M. tuberculosis* Lineage 1, which are referred as “ancestral” strains based on the deletion in their genome of the TbD1 region. Loss of TbD1 region is suspected to be responsible for the predominance of these lineages in current tuberculosis epidemiology (Brosch et al., 2002; Bottai et al., 2020). Furthermore, the observation reported here suggested that the 3,514-Da peak was absent in all TbD1-positive MBTC species. The TbD1 locus encompasses the m-pS6/mmpL6 operon, two proteins involved in the trans-inner-membrane transport of lipid residues. These proteins are absent and truncated in *M. tuberculosis* Lineages 2, 3, and 4, respectively. The 3,514 Da biomarker identified in MALDI-TOF spectra specifically for these lineages might then be hypothetically related to this proteomic difference. Mascot database searches (Matrix Science Inc.) were performed using the following parameters: UniProt database restricted to MBTC taxonomy, with formic acid as the digestion reagent selected and a mass shift tolerance of 2.0 Da on the *m/z* of 3,514 Da observed for the biomarker. However, this approach did not result in significant hits. Further studies will focus on the identification on the biomarker observed by 2-D electrophoresis gel separation and LC-MS/MS identification (Christner et al., 2014) to confirm the implication of the m-pS6/mmpL6 operon in the spectral differentiation of MBTC species.

This study does not present results obtained for *Mycobacterium caprae, Mycobacterium microti, Mycobacterium orygis*, and *Mycobacterium pinnipedii*, which are rare in human clinical diagnosis, and their epidemiology is considered location-dependent. In fact, a few dozen cases of human *M. microti* infections have been reported over the last few decades, and cases of human *M. caprae* infections were evaluated to represent less than 0.3% of human tuberculosis cases in Europe (Panteix et al., 2010; Prodinger et al., 2014). Although these numbers are considered underestimated because *M. microti* presents slow growth and is often undiagnosed and *M. caprae* is uneasily distinguished from *M. bovis*, the occurrence of these species in humans remains scarce compared to the occurrences of MBTC species presented here (*M. tuberculosis*, *M. bovis*, *M. bovis* BCG, and *M. africanum*), which were estimated at 9.5 million cases in the world in 2020 [World Health Organization [WHO], 2020 TB report]. Ecotypes that have never been reported in humans, such as *Mycobacterium mungi* and *Mycobacterium suricattae*, were also not included in this study. Therefore, the method may not be reliable for these species. However, as these variants are genetically closer to *M. bovis* and *M. africanum* than *M. tuberculosis* Lineages 2, 3, or 4 (Brites et al., 2018), the 3,514-Da biomarker might hypothetically be absent from their MALDI-TOF spectra.

The method proposed here relied on the observation of spectra containing or lacking specific biomarkers. This fast and low-cost method might be easily used by other laboratories already equipped with a MALDI-TOF-MS instrument to verify the identification of their isolates for clinical diagnostic purposes and in a retrospective manner for data already acquired on MBTC isolates. As discrimination described here was mostly driven by the absence/presence of the 3,514 Da peak, the direct observation of acquired spectra with software such as FlexAnalysis, which is provided with the MicroFlex device, limits the analytical skills needed for routine application, and the information regarding the absence/presence of biomarker can be obtained within minutes after spectra acquisition. The addition of the assessment of biomarker presence in the workflow of mycobacteria identification is an efficient and cost-reducing screening method, confirming the identification of an inquired isolate as *M. tuberculosis* or belonging to a less frequent member of the complex, suggesting the value of using an expensive sequencing method for identification purposes. Indeed, MALDI-TOF sample preparation and analysis, even with the addition of a data analysis step, remain cost- and time-limited. The mean consolidated cost of this method for a full target plate of 96 spots after considering all devices used (MALDI-TOF device, MALDI-TOF target plate, heat-block, stirrer, centrifuge, Fastprep device, and BSC), consumables, reagents, and staff occupation time was estimated at $3.93 for the identification of one isolate. Meanwhile, other mycobacteria identification methods costs are estimated at $1–2.3 for the anti-MPB64 assay (Toihir et al., 2011; Kanade et al., 2012) somehow without distinction inside the MBTC. Methods allowing distinction were estimated at $50 for genotyping MTBC (Singh et al., 2013) and $300–500 for WGS (Takiff and Feo, 2015), the latter two requiring several hours of experimentation. The applicability of the method for other laboratories is also consolidated by the correct classification rates obtained on isolates grown on different culture media routinely used in clinical mycobacteriology laboratories and by the observation of the specific biomarker independent of the medium used (94% for M7H10 medium, 87% for Coletsos medium). These results cannot be fully compared to the correct classification rate of 87.5% obtained for isolates grown on TransBK m4 agar medium due to the limited number of isolates used to assess the transposition of the method to other media (17 isolates grown on M7H10/Coletsos medium compared to 80 isolates grown on TransBK m4 agar medium).

Finally, as this work provides proof-of-concept by presenting preliminary results, variations in parameters such as the utilization of isolated growth in broth have not yet been investigated. The consistency of the discrimination described here on data generated on isolate growth in broth is of interest, as liquid medium results in a relatively quicker time of cultivation of mycobacteria than solid medium. A multicentric study performed in several countries, with different growth media (both liquid and solid) and MALDI-TOF devices, would confirm the reproducibility of this method for any routine laboratory. Sufficient data collection generated by this kind of study would be used to refine the model generated by the classifier algorithm and could allow further discrimination inside the MBTC.

The method presented here not only consisted of matching with a database, as reported for most previous studies using MALDI-TOF-MS identification of mycobacteria as a whole and MBTC in particular (Patel, 2015; Alcaide et al., 2018), but was completed with a method of biomarker-based discrimination. We postulate that this screening method is efficient, fast, and inexpensive that refines the identification within the MCTB, presenting a positive predictive value of 100%, indicating that the observation of identified peaks in spectra leads to the confirmation of the identification of isolates as *M. tuberculosis*.

## Data Availability Statement

Publicly available datasets were analyzed in this study. This data can be found here: https://www.mediterranee-infection.com/acces-ressources/donnees-pour-articles/reference-group-spectra_rapid-screening-method-for-identification-in-the-mycobacterium-tuberculosis/.

## Author Contributions

SR designed the experiments, analyzed and interpreted the data, and wrote the manuscript. JS and MM performed genomic analyses. ZH and FT collected samples and performed routine analyses. SB analyzed and validated the data, and wrote the manuscript. MD contributed to critically reviewing the manuscript, data interpretation, and validation. All authors declare that they have read and approved the manuscript.

## Conflict of Interest

MD is a co-founder and shareholder of Culture-Top, a startup whose TransBK m4 agar plate product is cited in this study. The remaining authors declare that the research was conducted in the absence of any commercial or financial relationships that could be construed as a potential conflict of interest.

## Publisher’s Note

All claims expressed in this article are solely those of the authors and do not necessarily represent those of their affiliated organizations, or those of the publisher, the editors and the reviewers. Any product that may be evaluated in this article, or claim that may be made by its manufacturer, is not guaranteed or endorsed by the publisher.

## Footnotes

1
https://www.mediterranee-infection.com

2 (https://www.mediterranee-infection.com/accesressources/donnees-pour-articles/reference-group-spectra_rapid-screening-method-for-identification-in-the-mycobacteriumtuberculosis/)
