# Supplementary material for: Rapid Identification of Mycobacterium tuberculosis Complex Using Mass Spectrometry: A Proof of Concept
Source: Front Microbiol. 2022 Mar 31;13:753969. doi: 10.3389/fmicb.2022.753969 (PMC9008353; doi:10.3389/fmicb.2022.753969)
Supplement: Supplementary file 1 [file Data_Sheet_1.docx]

**Supplementary Material**

**Rapid identification of *Mycobacterium tuberculosis* complex using mass spectrometry: a proof-of-concept.**

**Author: Simon Robinne ^1,2^, Jamal Saad ^1,3^, Madjid Morsli ^1,3^, Zelika Harouna Hamidou^1,3,4^, Fatah Tazerart ^3,5^, Michel Drancourt^1,2,3^, and Sophie Alexandra Baron^1,2,3*^**

**Table S1. Characteristic peak list used for quick-classifier algorithm 1 calculation.**

| *m/z* (Da) | DAve | PTTA | PAD | PWKW |
| --- | --- | --- | --- | --- |
| 3,514 | 19.69 | < 0.000001 | < 0.000001 | < 0.000001 |
| 2,386 | 6.82 | < 0.000001 | < 0.000001 | < 0.000001 |
| 5,500 | 12.51 | < 0.000001 | 0,00143 | < 0.000001 |
| 5,557 | 5.78 | < 0.000001 | 0,0339 | < 0.000001 |
| 5,517 | 25.07 | < 0.000001 | 0,0125 | < 0.000001 |
| 5,533 | 6.94 | < 0.000001 | 0,211 | < 0.000001 |
| 5,611 | 3.49 | < 0.000001 | 0,0254 | < 0.000001 |
| 3,787 | 14.66 | < 0.000001 | < 0.000001 | < 0.000001 |
| 1,0661 | 9.34 | < 0.000001 | < 0.000001 | < 0.000001 |
| 5,631 | 1.52 | < 0.000001 | 0,00849 | < 0.000001 |
| 5,496 | 10.3 | < 0.000001 | 0.225 | < 0.000001 |
| 1,0886 | 2.15 | < 0.000001 | < 0.000001 | < 0.000001 |
| 5,851 | 2.46 | < 0.000001 | < 0.000001 | < 0.000001 |
| 7,239 | 3.93 | < 0.000001 | < 0.000001 | < 0.000001 |
| 4,724 | 2.5 | < 0.000001 | < 0.000001 | < 0.000001 |
| 5,333 | 6.95 | < 0.000001 | < 0.000001 | < 0.000001 |
| 2,737 | 7.01 | < 0.000001 | < 0.000001 | < 0.000001 |
| 6,753 | 3.81 | < 0.000001 | 0.0000413 | < 0.000001 |
| 7,559 | 2.3 | < 0.000001 | < 0.000001 | < 0.000001 |
| 6,334 | 1.95 | < 0.000001 | 0.0047 | < 0.000001 |
| 6,375 | 9.32 | < 0.000001 | < 0.000001 | < 0.000001 |
| 6,993 | 0.87 | < 0.000001 | 0.0218 | < 0.000001 |
| 3,979 | 7.21 | < 0.000001 | < 0.000001 | < 0.000001 |

DAve: Difference between maximal and minimal average peak intensity among classes.

PTTA: *P* value from *t*-test/ANOVA

PAD: *P* value from Anderson-Darling test

PWKW: *P* value from Wilcoxon/Kruskal-Wallis test

**Table S2. Characteristic peak list used for quick-classifier algorithm 2 calculation.**

| m/z (Da) | DAve | PTTA | PWKW | PAD |
| --- | --- | --- | --- | --- |
| 3,515 | 17.81 | < 0.000001 | < 0.000001 | < 0.000001 |
| 2,386 | 4.31 | < 0.000001 | < 0.000001 | < 0.000001 |
| 3,787 | 8.54 | < 0.000001 | < 0.000001 | < 0.000001 |
| 5,517 | 17.32 | < 0.000001 | < 0.000001 | 0.0121 |
| 5,501 | 6.78 | < 0.000001 | < 0.000001 | 0.00152 |

DAve: Difference between maximal and minimal average peak intensity among classes.

PTTA: *P* value from *t*-test/ANOVA

PAD: *P* value from Anderson-Darling test

PWKW: *P* value from Wilcoxon/Kruskal-Wallis test
